# Terminal 10q26.12 deletion is associated with neonatal asymmetric crying facies syndrome: a case report and literature review

**DOI:** 10.1186/s13039-021-00554-1

**Published:** 2021-07-13

**Authors:** Qinghong Li, Chunmei Sun, Jinzhen Guo, Wen Zhai, Liping Zhang

**Affiliations:** 1 Department of Neonatology, Northwest Women’s and Children’s Hospital, Yanta District, No. 1616, Yanxiang Road, Xi’an, 7100061 Shaanxi People’s Republic of China; 2 Genetic Medical Center, Northwest Women’s and Children’s Hospital, Xi’an, 7100061 Shaanxi People’s Republic of China

**Keywords:** Asymmetric crying facies syndrome, Chromosome 10q26.12qter deletion, Genotype–phenotype correlation

## Abstract

**Background:**

The terminal 10q26 deletion syndrome is a clinically heterogeneous disorder without identified genotype–phenotype correlations. We reported a case of congenital asymmetric crying facies (ACF) syndrome with 10q26.12qter deletion and discussed their genotype–phenotype correlations and the potentially contributing genes involving the etiology of ACF.

**Methods and results:**

We reported a case of neonatal 10q26.12qter deletion and summarized the genotype–phenotype correlations and contributing genes of 10q26.12qter deletion from DECIPHER database and published studies. Meanwhile, we analyzed the potential pathogenic genes contributing to 10q26 deletion syndrome. The female preterm infant harboring 10q26.12qter deletion showed symptoms of abnormal craniofacial appearance with rare congenital asymmetric crying facies, developmental retardation, congenital heart disease, and pulmonary artery hypertension. The deleted region was 13.28 Mb in size as detected by G-banding and array comparative genome hybridization, containing 62 Online Mendelian Inheritance in Man (OMIM) catalog genes. We summarized data from 17 other patients with 10q26.12qter deletion, 11 from the DECIPHER database and 6 from published studies. Patients with monoallelic *WDR11* and *FGFR2* deletions located in 10q26.12q26.2 were predisposed to craniofacial dysmorphisms, growth retardation, intellectual disability and cardiac diseases.

**Conclusion:**

ACF is a facial dysmorphism frequently accompanied by other systemic deformities. It is a genetic abnormality that may associate with terminal 10q26.12 deletion. Early cardiac, audiologic, cranial examinations and genetic detection are needed to guide early diagnosis and treatment strategy.

## Background

10q26 deletion syndrome is a cytogenetic abnormality caused by the interstitial or terminal deletion of the long arm of chromosome 10. This abnormality was first described by Lewandowski et al. [1]*.* in 1978 and defined as 10q deletion syndrome by Wulfsberg et al*.* [2] in 1989. To date, approximately 100 cases of terminal 10qter deletions have been reported around the world [3]. The 10qter deletion syndrome has clinically heterogeneous symptoms that commonly characterized by growth and developmental retardation, intellectual disability, and craniofacial dysmorphisms. Recent studies added craniosynostosis, neurobehavioral disorders and hearing impairment symptoms to this genetic abnormality [3–5]. However, due to differences in the deleted fragment, no consensus has been achieved regarding the relations between deleted regions and symptoms of 10q deletion syndrome or the critical genes contributing to these dysfunctions. In this study, we report a case of 10q26.12qter deletion manifested with neonatal asymmetric crying facies (ACF) syndrome diagnosed based on clinical features and Giemsa-banding (G-banding) and array comparative genomic hybridization (aCGH) measures.

Congenital ACF is a rare condition that afflicts about 0.2%–0.6% of the infants. Infants with ACF present symmetric faces at rest and asymmetric faces while crying, which is usually caused by unilateral agenesis or hypoplasia of the depressor anguli oris muscle (DAOM) on one side of the mouth [6]. Approximately 45% to 70% of infants with ACF also exhibit other malformations [6]. These malformations most commonly involve the cardiovascular system, less frequently the respiratory system and rarely the central nervous system [7, 8]. When other disorders are involved, it is known as ACF syndrome [6]. The etiology of ACF syndrome has not been revealed until now. Our study reports a case of ACF syndrome associated with terminal 10q26.12 deletion. It summarizes the possible relations between 10q26.12 and congenital abnormities, as well as the potential target genes.

### Patient information

A female infant was born preterm at 34 gestational weeks by cesarean section with a birthweight of 1480 g (< 3rd percentile), length of 40 cm (3rd–10th percentile), and head circumference of 28 cm (3rd percentile). The Apgar scores at 1 and 5 min were 8 and 9, respectively. No asphyxia, hypoxia, or respiratory distress were found at delivery. She was the first child of healthy, unrelated parents, a 24-year old father and mother of the same age. Neither parent had a family history of intellectual disability, behavioral difficulties, or malformations. Her mother experienced an human papilloma virus (HPV) infection about six months before becoming pregnant via natural conception, and an HPV recurrence in the late stages of her pregnancy. She developed total placenta previa and was diagnosed with fetal growth restriction at 28 gestational weeks. She also experienced grade II amniotic fluid contamination during delivery. A Down syndrome screening test and oral glucose tolerance test did not show any abnormities, and no subsequent amniocentesis or peripheral blood extraction for chromosome or DNA detection was performed. B ultrasound showed a 1.5 mm of nuchal translucency thickness at 11 gestational weeks, and no obvious abnormities were shown in further ultrasonic images at appropriate gestational weeks.

### Diagnostic assessment

**Fig. 1:**
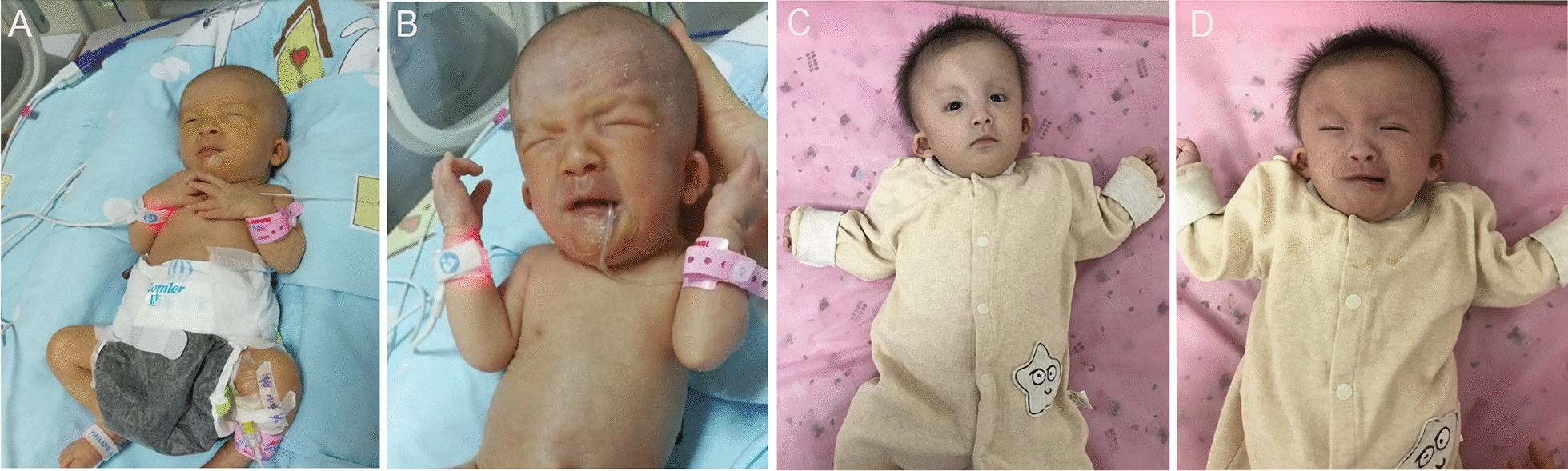
Craniofacial appearance of the infant at rest or on crying 15 days or 6 months after birth. **a**, **b** appearance at rest or on crying 15 days after birth; **c**, **d** appearance at rest or on crying 6 months after birth

The infant presented with an asymmetric facial expression when crying with the right side of her mouth deviating inferior-laterally when crying but was symmetrical at rest (Fig. 1). She also presented with other facial abnormalities, such as pale skin, bilateral thick overfolded helices, deep eye sockets, small mouth opening, and high palatal arch. Blood tests showed no abnormalities at birth. Re-examination at 2 days after birth showed lower hemoglobin (100 g/L) and platelets (17 × 10^9^/L). She exhibited poor oral feeding due to weak sucking and swallowing, as well as uncoordinated suck-swallowing. The in-hospital physical examinations showed hypotonia and weak prepotential reflexes. The neonatal hearing screening and chest-abdomen X-ray found no abnormalities. A cardiac ultrasound examination showed an atrial septal defect (diameter of 3 mm), a patent foramen ovale (diameter of 2 mm), and patent ductus arteriosus (diameter of 2.5 mm). Cerebral magnetic resonance imaging (MRI) showed a patchy T1/T2 signal in the white matter of the bilateral lateral ventricle, and abnormally low T1 and T2 signals on the back side of the left cerebellar hemisphere. Therefore, mild brain injury and focal hemorrhage in the left cerebellar hemisphere were considered. Based on the craniofacial appearance and other systemic manifestation, a congenital disease with chromosome abnormality or gene mutation was suspected.

**Fig. 2:**
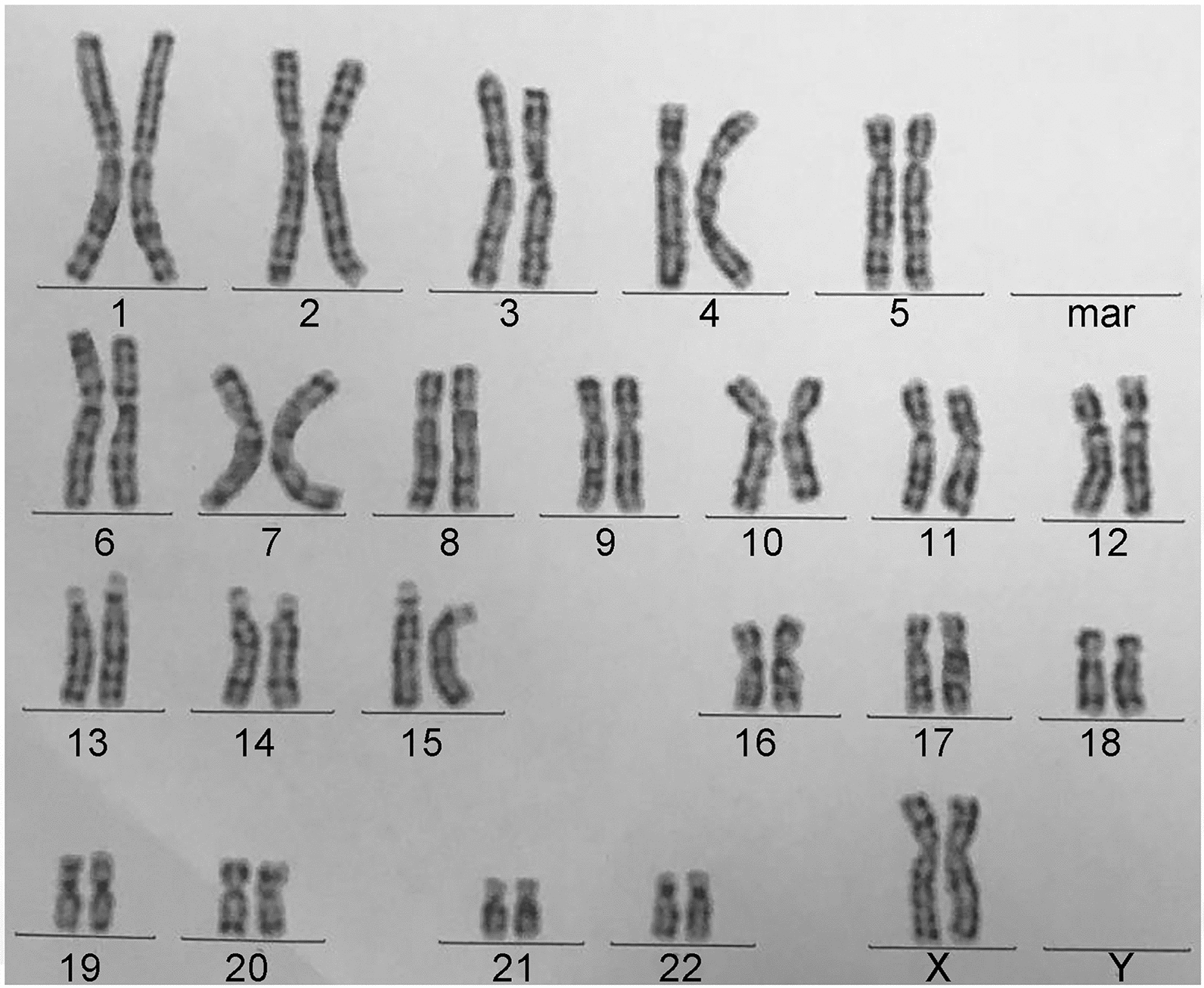
The G-banded karyotype of the cultured peripheral blood cells isolated from the proband. The proband showed a loss of 10q with a karyotype of 46,XX,del(10)(q26)

**Fig. 3:**
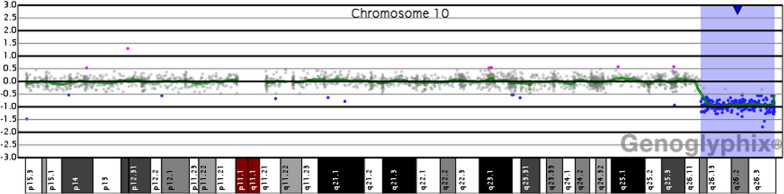
Oligonucleotide-based aCGH analysis using peripheral blood cells showed a 13.49-Mb deletion in 10q26 [chr10:122119070 to 135403394]

**Fig. 4:**
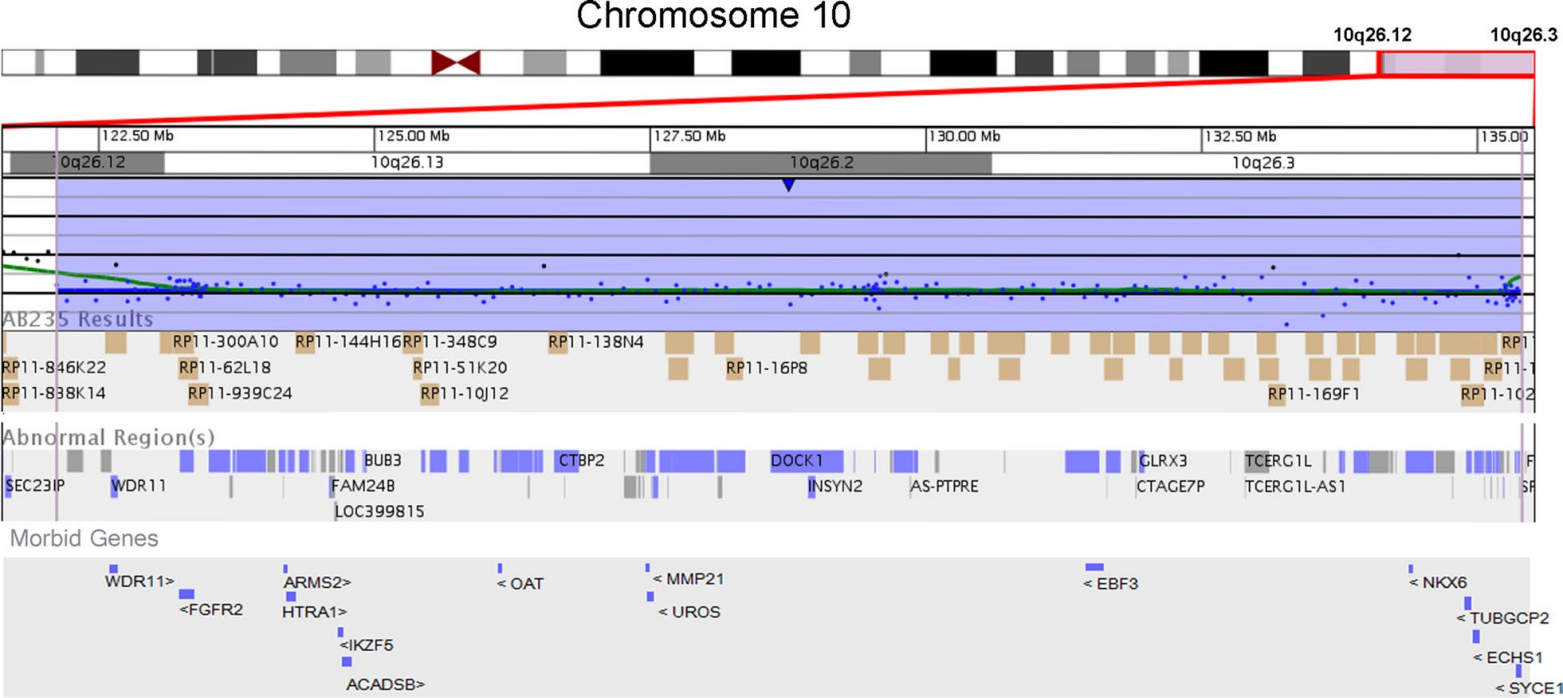
Schematic overview of the chromosome 10q26.12-q26.3 region according to UCSC genome browser [http://genome.ucsc.edu]

To further assess whether the proband had a de novo or inherited genome abnormality, a conventional karyotyping was performed. After obtaining written informed consents, peripheral blood samples were extracted from the infant and her parents. A minimum of 20 metaphase cells were analyzed from peripheral blood cell cultures stimulated with phorbol-12-myristate-13-acetate for 72 h. G-banding was performed at a resolution of 400–550 bands per haploid set. The results revealed that both parents presented normal G-banded chromosomes, but the infant showed a loss of the chromosomal fragment at the end of the long arm of one chromosome 10 with a karyotype of 46,XX,del(10)(q26) (Fig. 2). A de novo genome abnormality was suspected since the parents had normal phenotypes and karyotypes. However, we could not exclude the inherited risk of balanced parental abnormality due to the lack of fluorescence in situ hybridization detection data. The high sensitive oligo a-CGH analysis was performed to identify the deleted fragment of the proband by extracting DNA from peripheral blood cells and detecting using a customized CGX™ 8 × 60 K oligo microarray with a resolution of ~ 190 kb in the backbone and ~ 28 kb in the targeted regions (PerkinElmer, MA, USA). Microarray data were extracted and visualized using Genoglyphix software (PerkinElmer). The genomic positions were determined using GRCh37/hg19, UCSC Genome Browser. The data confirmed a 13.49 Mb deletion at 10q26.12qter (122,119,070 to 135,403,394 bp) (Fig. 3). The deleted fragment contained 106 RefSeq genes and 62 of them were OMIM genes (Fig. 4). The patient was finally diagnosed of neonatal asymmetric crying facies syndrome with 10q26.12qter microdeletion based on the her clinical features and the genomic detections.

### Therapeutic intervention

The infant was admitted to the neonatal intensive care unit for preterm labor and irregular breath care. She was treated with red blood cell suspension infusion, plasma transfer and oxygen supplementation. Nasal feeding was administrated due to poor oral feeding until reaching sufficient milk intake. The infant was discharged 18 days after delivery.

About 5 months later, a cardiac ultrasound examination indicated that the diameter of the atrial septal defect was 2.1 mm, the patent foramen ovale was 1.4 mm, and the patent ductus arteriosus was 4 mm. Pulmonary arterial hypertension was measured at approximately 74 mm Hg. Therefore, the infant underwent cardiac surgeries for transcatheter closure of the atrial septal defect and patent foramen ovale, as well as ligation of the patent ductus arteriosus. The cerebral MRI follow-up at 8 months showed that the abnormal signal behind the left cerebellar hemisphere had disappeared, and the white matter myelination matched characteristics of infants at 5–6 months of age.

### Follow-up and outcome

The infant exhibited motor developmental retardation. She could raise her head only temporarily and was unable to turn over her body, sit, or crawl independently at 8 months. She achieved a total score of 12.12 [95% CI: 5.55–18.69] in the gross motor function test. At 31 weeks after birth, the infant had gross and fine motor performance equivalent to 4-week old and 13-week old infants, respectively, the adaptive behavior to 9-week old, the linguistic performance to 8-week old, and the individual social skills to 12-week old infants. These Gesell test results suggested a global development retardation. The infant received special physical training for gross motor functions, including head control and sitting and crawling exercises, and her head control improved. Meanwhile, occupational and speech therapies were also initiated.

## Discussion

### 10q26 deletion associated clinical symptoms

The development and application of high-resolution microarray platforms have enabled the precise delineation of 10q26 deletion associated symptoms in clinical practice. Ramons et al*.* summarized 23 cases of distal 10q26 deletions that were diagnosed by array testing, with deleted regions ranging from 3.5 to 17.4 Mb [9]. However, no clear correlation has been delineated between the size and the location of the deleted 10q and the severity of the phenotypes. Our report highlights that congenital ACF syndrome is associated with 10q26.12 deletion.

Previous studies have concluded that the 10q deletion syndrome is exclusively accompanied by developmental retardation, intellectual disability, and craniofacial abnormities, despite a wide array of other clinical characteristics. In addition to these common features, a considerable phenotypic heterogeneity have been observed even in patients belonging to the same family and sharing the same deletion [3]. We searched the DECIPHER and Pubmed databases [3, 4, 10–13] for cases with 10q26.12qter deletion and identified 17 patients (11 in DECIPHER and 6 in Pubmed; 11 females and 6 males) (Table 1). Only one patient was diagnosed with inherited chromosomal abnormality, whereas twelve were de novo and four were of unknown origins. All deleted regions spanned from 10q26.12 to the end of the chromosome, with a deletion length between 10.28 and 14.08 Mb. Facial dysmorphic features, developmental retardation, and cardiac defects were three leading symptoms among these patients. Twelve (70.6%) cases presented with facial dysmorphic features and four (22.2%) with microcephaly. Six (33.3%) cases presented with eye defects, such as strabismus, blepharophimosis and cataract. Six (33.3%) patients presented with ear abnormalities, such as low-set malformed ears, abnormality of the helix, sensorineural hearing loss. Cardiac diseases were reported in seven (38.9%) cases, cerebral aplasia/hypoplasia in five (27.8%), and renal or urinary tract pathogenesis in five (27.8%). Global developmental retardation was found in most of the patients (88.9%) except two.

**Table 1:** Summary of clinical features of terminal 10q26.12 deletion in patients identified from DECIPHER database and Pubmed

DECIPHER ID and reported cases	Sex/age	Deletion areas	Deletion size (Mb)	Inheritance	Phenotypes						
					1	2	3	4	5	6	7
Current case	F/ < 1 m	10:122119070 –135403394	13.28	De novo	+	−	+	+	+	+	+
274013	M/11 y	10:121355765–135439229	14.08	De novo	+	−	−	−	−	+	+
330947	F/9 y	10:121468628–135426384	13.96	NA	+	+	−	−	−	−	+
398749	M/ < 12 m	10:122736421–135430043	12.69	uncertain	+	−	−	+	+	+	−
324337	M/2y	10:122862637–135434178	13.57	De novo	+	−	+	+	−	+	+
285689	M/NA	10:122761628–135372492	12.61	De novo	+	+	+	−	−	−	+
304635	F/37 y	10:123241651–135404523	12.16	NA	−	−	−	−	−	−	+
385190	F/12 m	10:123393359–135434149	12.04	De novo	−	−	−	−	−	−	+
285837	F/28y	10:124347712–135372492	11.02	De novo	−	−	+	−	−	+	+
283660	F/ < 12 m	10:124500982–135404471	10.9	Maternally inherited	−	+	−	−	−	−	+
285822	M/4y	10:124785795–135372492	10.59	De novo	+	−	−	−	−	−	+
300399	F/12 m	10:125121038–135404523	10.28	NA	−	−	+	−	−	−	+
Chang 2013	F/10.5 y	q26.12–q26.3	NA	De novo	+	−	+	+	+	−	+
Vera-Carbonell 2015	F/17 y	10:122014670–135404523	13.5 Mb	De novo	NA	−	−	−	−	−	+
Faria 2016	F/1 m	10:122095511–135203489	13.1 Mb	De novo	+	−	−	+	+	−	−
Lin 2016	F/5 y	10:122387570–135427143	13.04 Mb	De novo	+	−	−	−	+	−	+
Gunnarsson 2009	F/Newborn	10:122736794–135259604	12.5	De novo	+	+	−	−	+	−	+
Miller 2009	M/4.5 y	10:122970216–135434303	12.4 Mb	De novo	+	−	−	+	+	−	+

*F* female, *M* male, *NA* not available, *m* month, *y* year

Phenotypes:

1-Craniofacial dysmorphisms

2-Microcephaly

3-Ophthalmic defects

4-Abnormal ears and/or hearing impairment

5-Congenital heart defects

6-Cerebral aplasia/hypoplasia

7-Global developmental delay

In our case, she presented with global developmental delays, cardiac defects, facial abnormalities, and hypotonia. Most of her clinical signs were similar to those patients with pure 10q26 deletions as previously characterized through high-resolution molecular techniques. Our patient also presented with symptoms of congenital ACF syndrome, which was caused by unilateral agenesis or hypoplasia of the DAOM or depressor labii inferioris muscle (DLIM) and the resultant lower-lip depression of the intact side. However, her face was symmetrical at rest. The ACF symptom was not mentioned in other studies involving 10q26.12 deletion.

### Potential etiology of neonatal asymmetric crying facies syndrome

Neonatal ACF, also termed as congenital unilateral lower lip palsy, is characterized by the absence or weakness of the downward motion of the lateral side of the mouth with crying [14]. The downward motion of the lower lip is controlled by the coordinated action of four muscles (DAOM, DLIM, mentalis muscle and platysma muscle) innervated by the facial nerve branches. ACF are preferably caused by the hypoplasia of the DAOM and less commonly the DLIM. Trauma caused injury to the peripheral branches of the facial nerve may also result in this deformity in rare cases [15].

Up to now, the etiologies and molecular mechanisms of ACF syndrome have been unclear. Previous studies documented an infant with ACF syndrome who was born to a mother with a six-year history of diabetes, and the other two born to each mother with gestational diabetes [8, 16]. It has been speculated that intrauterine retinoic acid exposure, as well as embryologic fault of the DAOM or the mandibular branch of the facial nerve, also may account for the etiology of ACF syndrome [17]. Besides, pediatrists also associate neonatal ACF with increased HBeAb and HBcAb adopted from mother infected with hepatitis B virus [6]. Several studies associate the occurrence of ACF syndrome with the deletion of 22q11.2 or 4p. Pasick et al*.* found that 14% of the subjects (117/836) carrying 22q11.2 deletions exhibited ACF [18]. However, the genetic contributions of the genes located in 22q11.2 to symptoms of ACF remain unexplained.

We found that terminal 10q26.12 deletion was associated with ACF syndrome. The genotype–phenotype correlation of 10q deletion has been extensively studied, and some connections have been established. However, the exact correlation between genotype and phenotype remains to be elucidated. It is acknowledged for some of these syndromes that multiple genes may be implicated in the duplicated or deleted region, but only one of them is gene-dosage sensitive and causative for the specific clinical signs [19]. The identified microdeletion in this proband involves 106 known genes and 62 of them are OMIM genes. Fourteen OMIM genes are underlined to be morbid genes from DECIPHER database in case of 10q26.12qter deletion (Table 2). Several compelling studies reported that interstitial deletion at 10q26.2q26.3 or terminal 10q26.2 deletion only caused mild neurocognitive phenotypes or minor dysmorphic features with near normal development and intellect [20–23]. Therefore, we speculate that the deleted genes located at 10q26.12q26.13 (*OAT*, *ACADSB, IKZF5, HTRA1, ARMS2, FGFR2 and WDR11*) are responsible for those notable malformations. Of them, *WDR11* and *FGFR2* are confirmed monoallelic genes, which may increase susceptibility to the genomic disorder partially due to the haploinsufficiency of these genes. It should be noted that the number of haploinsufficient genes in deleted regions might be the critical contributor to the phenotypes of 10q26 deletion. However, there is insufficient evidence on the pathogenicity of haploinsufficiency for the candidate genes and their genotype–phenotype correlations with 10q26 deletions.

**Table 2:** Genes in the region of 10q26.12qter and the associated diseases

Genes	Location	OMIM	Description	DDG2P	%HI	pLI	Diseases
SYCE1	10:135367404–135382876	611486	Synaptonemal complex central element protein 1	NA	74.27	0.00	Premature ovarian failure, Spermatogenic failure
ECHS1	10:135175984–135187193	617817	Enoyl-CoA hydratase, short chain 1	Confirmed: Biallelic	61.36	0.00	Pachygyria, microcephaly, developmental delay, and dysmorphic facies, with or without seizures
TUBGCP2	10:135093135–135125841	618737	Tubulin gamma complex associated protein 2	Probable: Biallelic	45.69	0.00	NA
NKX6-2	10:134598297–134599556	605955	NK6 homeobox 2	Probable: Biallelic	53.68	0.03	Spastic ataxia 8, autosomal recessive, with hypomyelinating leukodystrophy
EBF3	10:131633547–131762105	607407	EBF transcription factor 3	Confirmed: Monoallelic	3.89	1.00	Hypotonia, ataxia, and delayed development syndrome
UROS	10:127477146–127511817	606938	Uroporphyrinogen III synthase	Confirmed: Biallelic	69.16	0.03	Porphyria, congenital erythropoietic
MMP21	10:127455022–127464390	608416	Matrix metallopeptidase 21	Confirmed: Biallelic	82.02	0.00	Visceral heterotaxy, 7, autosomal
OAT	10:126085872–126107545	613349	Ornithine aminotransferase	NA	31.64	0.00	Gyrate atrophy of choroid and retina with or without ornithinemia
ACADSB	10:124768495–124817827	600301	Acyl-CoA dehydrogenase short/branched chain	NA	65.42	0.00	NA
IKZF5	10:122990805–123008825	619130	IKAROS family zinc finger 5	NA	23.98	0.78	Thrombocytopenia
HTRA1	10:124221041–124274424	602194	HtrA serine peptidase 1	NA	19.96	0.00	Macular degeneration, age-related, neovascular type; Cerebral arteriopathy, with subcortical infarcts and leukoencephalopathy, type 2
ARMS2	10:124214169–124216868	613778	Age-related maculopathy susceptibility 2	NA	97.47	0.00	Macular degeneration, age-related, 8
FGFR2	10:123237848–123357972	176943	Fibroblast growth factor receptor 2	Confirmed: Monoallelic	0.50	1.00	Antley-Bixler syndrome without genital anomalies or disordered steroidogenesis; Apert syndrome; Beare-Stevenson cutis gyrata syndrome; Bent bone dysplasia syndrome; Craniofacial-skeletal-dermatologic dysplasia; Scaphocephaly, maxillary retrusion, and mental retardation
WDR11	10:122610687–122669036	606417	WD repeat domain 11	Confirmed: Monoallelic	39.49	0.00	Hypogonadotropic hypogonadism with or without anosmia, cardiac anomaly and growth retardation

WDR11 is a highly conserved protein that contains twelve WD domains. Genetic studies have revealed that *WDR11* contributed to the occurrence of congenital hypogonadotropic hypogonadism and Kallmann syndrome [24]. Sutani et al*.* identifies *WDR11* as another causative gene for cardiac anomaly and severe growth retardation in 10q26 deletion syndrome [25]. FGFR2 is a receptor tyrosine kinase that regulates a wide range of biological processes during development or in adult tissues [26]. Majority of missense mutations lead to constitutive activation of FGFR2 and its downstream molecular pathways [27]. *FGFR2* is marked by a high probability of being loss of function intolerant (pLI = 1) and associates with a wide array of pathological and clinical features, including craniofacial-skeletal-dermatologic dysplasia, scaphocephaly, maxillary retrusion, and mental retardation. Besides, Choucair et al*.* identified *FGFR2*, *NSMCE4A*, and *ATE1*, three consecutive genes located in 10q26.12, to be candidates for facial dysmorphism, growth cessation, and heart defects, respectively [28]. Therefore, *WDR11* and *FGFR2* deletions might be responsible for the clinical characteristics of the present case, such as global developmental delays, cardiac defects and facial abnormalities. However, whether *WDR11* and *FGFR2* deficiencies result in agenesis or hypoplasia of the DAOM, require further investigations. Meanwhile, less evidence revealed the associations between other morbid genes (*OAT*, *ACADSB, IKZF5, HTRA1* and *ARMS2)* and symptoms of the present case.

In addition to the supposed genes located in the critical regions of 10q26.12q26.2, the contributions of genes outside this region could not be fully excluded, such as *EBF3* and *ECHS1*. EBF3 is a member of the collier/olfactory-1/early B -cell factor family of proteins that are required for neuronal specification, axon guidance, and dendritogenesis during nervous system development [29]. Previous studies delineate that haploinsufficiency of EBF3 is a critical contributor to developmental delay, intellectual disability, and particular facial dysmorphisms in patients bearing point mutations and indels in this gene [29, 30]. Mutations in *ECHS1* cause rare autosomal recessive disorders mainly presenting with developmental delay, dystonia, feeding difficulties, and abnormal neuroimaging with bilateral basal ganglia involvement [31]. *ECHS1* deficiency is also linked with the occurrence of pachygyria, microcephaly and dysmorphic facies.

### Management of neonatal asymmetric crying facies syndrome

The current treatment for ACF syndrome aims to correct cosmetic and functional deficits and to improve patient’s quality of life. Minimally invasive chemodenervation is a therapeutic strategy that improves smile symmetry and lower-lip position by injecting lidocaine and botulinum toxin A into the contralateral DAOM. The procedure has led to a 4-month improvement in smile symmetry [32]. Permanent strategies for correcting ACF asymmetry consist of selective myectomy of the contralateral DAOM and selective neurectomy of the contralateral marginal mandibular branch of the facial nerve [18].

No treatment may be urgently required for neonatal ACF patients with isolated facial anomalies if the cosmetic problems are minor. However, as described in previous studies, other malformations often accompany ACF feature [6]. Rai et al*.* advise pediatricians to be aware that this mild facial anomaly may be associated with severe internal organ system anomalies, most commonly cardiac-related [33]. Some authors advocate that ACF should be considered a marker of congenital malformations [34]. For neonate presenting with ACF, early cardiac, audiologic, cranial and ocular examinations as well as genetic detection are recommended to guide early diagnosis and treatment interventions. For neonatal ACF involving other systems, tolerable surgeries, medical treatments and physical training should be considered as early as possible.

## Conclusions

Our study reported a case of neonatal ACF syndrome accompanied by global developmental delay, cardiac defects, and facial dysmorphisms. A 13.49 Mb terminal deletion of 10q26.12 involving 62 OMIM genes was confirmed by karyotyping and aCGH. We supposed that terminal 10q26.12 deletion is associated with phenotypes of ACF syndrome and that *FGFR2* and *WDR11* might be candidate genes implicated in this disorder. However, the causal relation between 10q26 deletion and the ACF syndrome phenotype should be examined further to identify the contributing genes and mechanisms contributing to this syndrome.

## Data Availability

The datasets used during the present study are available from the corresponding author upon reasonable request.

## Abbreviations

OMIM: Online Mendelian Inheritance in Man
ACF: Congenital asymmetric crying facies
G-banding: Giemsa banding
aCGH: Array comparative genomic hybridization
HPV: Human papilloma virus
DAOM: Depressor anguli oris muscle
DLIM: Depressor labii inferioris muscle
DECIPHER: Database of Chromosomal Imbalance and Phenotype in Humans using Ensembl Resources
MRI: Magnetic resonance imaging
